# Self-management of blood pressure following hypertensive pregnancy (the SNAP2 trial): a prospective, multi-centre, unblinded, randomised controlled trial protocol

**DOI:** 10.1186/s12872-026-06359-7

**Published:** 2026-08-04

**Authors:** Lucy Goddard, Ellie Newbury, Katherine Tucker, Natalie Barry, Debra Bick, Helen Campbell, Lucy C Chappell, Paphapisa Chotthanawathit, Kate Corfield, Marcus Green, Pankaj Gupta, Lisa Hinton, Sandra Ignew, Nicola Kenealy, Paul Leeson, Rosa Mackay, Lucy Mackillop, Christine McCourt, Kate Moon, Jenny Myers, Cynthia Ochieng, Hannah Rayment-Jones, Oliver Rivero-Arias, Cristian Roman, Milensu Shanyinde, Mayue Shi, Lionel Tarassenko, Lucy Yardley, Ly-Mee Yu, Richard J. McManus

**Affiliations:** 1 Nuffield Department of Primary Care Health Sciences, University of Oxford, Oxford, United Kingdom; 2 Primary Care Clinical Trials Unit, Nuffield Department of Primary Care Health Sciences, University of Oxford, Oxford, United Kingdom; 3 Maternal and Fetal Health Research Centre, University of Manchester, Manchester, United Kingdom; 4 Warwick Clinical Trials Unit, Warwick Medical School, University of Warwick, Coventry, United Kingdom; 5 National Perinatal Epidemiology Unit, Nuffield Department of Population Health, University of Oxford, Oxford, United Kingdom; 6 Department of Women and Children’s Health, Kings College London, London, United Kingdom; 7 Action on Pre-eclampsia charity, Worcestershire, United Kingdom; 8 Department of Cardiovascular Sciences, Leicester British Heart Foundation Centre of Research Excellence, University of Leicester, Leicester, United Kingdom; 9 The Motherhood Group, London, United Kingdom; 10 Cardiovascular Clinical Research Facility, RDM Division of Cardiovascular Medicine, University of Oxford, Oxford, United Kingdom; 11 Nuffield Department of Women’s and Reproductive Health, University of Oxford, Oxford, United Kingdom; 12 School of Health and Medical Sciences, City St George’s, University of London, London, United Kingdom; 13 School of Psychological Science, University of Bristol, Bristol, United Kingdom; 14 Institute of Biomedical Engineering, Department of Engineering Science, University of Oxford, Oxford, United Kingdom; 15 NIHR Oxford Biomedical Research Centre, Oxford, United Kingdom; 16 School of Psychology, University of Southampton, Southampton, United Kingdom; 17 University of Brighton and University of Sussex, Brighton and Sussex Medical School, Brighton, United Kingdom

**Keywords:** Hypertensive disorders of Pregnancy, Postnatal hypertension, Self-management and self-monitoring, Secondary to primary care transition

## Abstract

**Background:**

Hypertensive disorders of pregnancy affect around 10% of pregnant women and can continue following childbirth. Such hypertension that persists postnatally can be unpredictable and may have long-term health consequences but care can be sub-optimal with poor support as women transition from maternity to community services. However, there is little evidence to guide best practice for managing postnatal hypertension, and these gaps in evidence and care disproportionately affect women from minoritised ethnic and socioeconomically disadvantaged backgrounds.

Previous work suggests self-management where women self-monitor blood pressure (BP) and self-titrate medication, overseen by clinicians, could improve BP control in the short and long term but has been limited to single geographical areas. The aim of the SNAP2 trial is to evaluate the clinical and cost effectiveness of a self-management intervention at scale.

**Methods:**

SNAP2 is a multi-centre unblinded randomised controlled trial. A planned sample of at least 628 participants across England with any pregnancy hypertension will be randomised within 7 days post birth to a digitally-supported self-management intervention, including self-monitoring of BP and clinician-guided titration of anti-hypertensive medication, or usual care. A cross-cutting theme is evaluating how health equity can best be ensured in postnatal hypertension management.

The primary outcome is the difference in mean daytime ambulatory diastolic BP between intervention and usual care, measured between 22 to 39 weeks postpartum. Secondary outcomes include other measures of BP, maternal quality-of-life, medication adherence, adverse outcomes and readmission to hospital up to one year after birth. A qualitative process evaluation will explore how the intervention is implemented and integrated into women’s lives and clinical pathways. An economic analysis will evaluate within-trial cost and outcomes and long-term economic modelling.

**Discussion:**

The SNAP2 trial will provide essential data on the clinical and cost-effectiveness of a self-management intervention that, if proven effective, could improve current sub-optimal postnatal care for BP management. In-depth qualitative and equity data will provide important insight to inform potential implementation.

Throughout the manuscript, in our references to women, we encompass all birthing people.

**Clinical trail number:**

https://doi.org/10.1186/ISRCTN11042045.

**Supplementary Information:**

The online version contains supplementary material available at https://doi.org/10.1186/s12872-026-06359-7.

## Background

Hypertensive disorders affect 8–10% of all pregnancies and are a leading cause of maternal morbidity and mortality worldwide [1, 2]. High blood pressure (BP) that persists postnatally is dangerous, unpredictable, and may be life-threatening, leading to an increased risk of hypertension and cardiovascular disease for at least the next 20 years, even if BP returns to normal following birth [3, 4].

Exacerbating long-term cardiovascular risk, inequalities in access to timely diagnosis, treatment, and follow-up care disproportionately affect women from minoritised ethnic groups and those living in areas of higher deprivation [5, 6]. Furthermore, MBRRACE reports highlight that women from Black, Asian and other minority ethnic backgrounds, as well as those living in the most deprived areas, face higher risks of severe complications and mortality associated with hypertension during and after pregnancy [7].

Care following postnatal discharge is often haphazard and sub-optimal due to the transition from maternity to primary care services and competing demands for both women and clinicians [8–13]. Therefore, hypertension management following pregnancy presents a unique timepoint which differs from general essential hypertension management [14]. Importantly, current national and international guidance for post-partum hypertension lacks robust, high-quality data that addresses existing disparities to inform care [15].

Previous studies carried out by this group have demonstrated sustained reductions in BP when postnatal women self-manage BP (using digitally-enabled interventions with daily self-monitoring to prompt timely medication titration) compared to usual care, which appear independent of antihypertensive treatment and persist for up to 3.5 years [16–18]. Most recently, a study involving 220 women from one hospital site found that physician-guided self-management of outpatient postnatal blood pressure, following hypertensive pregnancy, was associated with a 6.5 (95% CI 4.2,8.8)/5.8 (4.2, 7.4) mmHg lower (systolic/diastolic) lower ambulatory blood pressure at 9 months along with favourable cardiac remodelling compared to usual care [18]. These interventions were safe, appeared to reduce re-admissions and participants reported enhanced sense of control and a reduction in blood pressure-related anxiety [18, 19].

These data suggest that optimising BP control in the immediate post-partum weeks may represent an intervention within a critical time window, with potential long-term cardiovascular benefits [20]. However, both previous studies were relatively small-scale, in one geographical area, and relied on small numbers of clinicians to deliver them. Innovative postpartum interventions that can be scaled up are required to optimise BP management to enhance cardiovascular disease prevention [21, 22].

Subsequently, based on the previously trialled intervention, a new version has been co-designed in collaboration with a diverse group of women with lived experience of hypertension during pregnancy and the postnatal period [23]. The aim of the SNAP2 trial is to test the clinical and cost-effectiveness of the complex digitally-supported self-management intervention to enable self-monitoring of blood pressure and titration of antihypertensive medication by women following a hypertensive pregnancy.

## Methods: participants and interventions

This is an unblinded prospectively registered, multi-centre randomised controlled trial with embedded internal pilot, economic analyses and process evaluation [ISRCTN11042045]. It will take place at 25–30 maternity units across England.

This protocol is reported according to the SPIRIT checklist [24] (Supplementary file_CompletedSPIRITchecklist).

### Participants

Women with any type of pregnancy hypertension (including gestational hypertension, pre-eclampsia and chronic hypertension), requiring anti-hypertensive medication in the postnatal period (Table 1) will be randomised to one of two treatment arms: usual care, or usual care + self-management of blood pressure.

**Table 1:** SNAP2 trial inclusion and exclusion criteria

Inclusion criteria	Exclusion criteria
Participant with pregnancy hypertension including: • chronic/essential hypertension (predating current pregnancy or requiring treatment before 20 weeks gestation), • gestational hypertension (new-onset hypertension from 20 of index pregnancy) • pre-eclampsia (proteinuria or other metabolic changes following chronic or gestational hypertension)	Participant does not wish to self-monitor/self-manage their blood pressure.
Still requiring antihypertensive medication at randomisation following delivery	Participant already taking part in another trial that might affect their anti-hypertensive prescription.
Within 7 days following birth.	
Participant is willing and able to give informed consent for participation in the study and to comply with trial requirements.	
Aged 18 years or above.	
Willing to allow their primary and secondary healthcare teams, if appropriate, to be notified of participation in the trial.	
Access to a smartphone compatible with the app (including provision by trial team).	

#### Usual care group

Women randomised to usual care will continue to receive routine postnatal care as available in their area, most likely overseen initially by the obstetric and midwifery teams, with subsequent transfer to their General Practitioner (GP) (family doctor).

#### Self-management group

Women randomised to the self-management intervention will be provided with a validated BP monitor and taught how to self-monitor their blood pressure. They will be asked to self-monitor every day until their blood pressure is stable, then weekly thereafter until the primary outcome is collected at 6 months.

Participants will be given access to a bespoke smartphone app (My BP Care, iOS and Android, developed by the research group) through which they will submit blood pressure readings, and new medication information [23]. The My BP Care app and associated electronic systems (data transmission and storage, clinician dashboard and automated messaging) have been iteratively designed in-house by a team from Institute of Biomedical Engineering in the University of Oxford. These systems and all other elements of this “complex intervention” including supporting leaflets, app messages and email content, have been co-designed, piloted and refined with extensive user testing and feedback, prior to commencement of the trial [16, 18, 23]. The app communicates blood pressure readings to clinical teams and facilitates communication of medication changes, but is not considered a medical device.

##### Participants

Blood pressure readings will be taken twice each day, 1 min apart and ideally first thing in the morning and entered by the participants into the My BP Care app. The second reading will be used to inform the required action following the process in our previous trials [16, 18]. If a very high (Systolic ≥ 150 mmHg or Diastolic ≥ 100 mmHg) or very low (Systolic < 100 mmHg and Diastolic < 80 mmHg) reading is entered, a third BP reading is automatically requested via the app. When participants enter a BP reading requiring a medication review, they will be prompted via the app to contact their maternity team if within 14 days of birth, or their GP thereafter. Appropriate timelines for action, including if experiencing symptoms, will be provided to the participants though automated app messaging with out-of-hours contact details given (supplementary file – SNAP2_BloodPressureCareLeaflet_V2.0_28Mar2024). Once blood pressure has settled to a steady state (in participants with chronic hypertension) or normalised (in those with gestational hypertension/pre-eclampsia), the cadence for BP readings will reduce to weekly. If a persistently abnormal reading is taken during weekly monitoring, the participant will be prompted to switch back to daily readings until BP stabilises again. Medication adjustments will be based on NICE recommendations [22, 25].

A supporting leaflet will be given to each participant, including (a) instructions on how to take blood pressure readings and how to enter readings into the app; (b) a colour-coded table (red, amber, green) showing the action required following abnormal readings; and (c) relevant contact numbers (Appendix -BloodPressureCareLeaflet_V2.0_28Mar2024). Regular automated reminders and motivational messages will also be sent via the app.

##### Clinicians

Supervising clinicians will have access to the readings submitted via a web-based dashboard. In conjunction with this, in the case of abnormal readings, clinicians will receive an email prompting details (study ID, days postpartum, last 3 BP readings, last reported medication) of the required medication review. Medication guidance on up and down titration based on NICE recommendations for management of hypertension in pregnancy will inform the medication changes for each participant [22, 25]. Medication adjustment will be communicated to participants via the app and SMS if delivered via the My BP Care Clinician Dashboard, or by usual methods of communication if via the standard systems of the participant’s maternity unit or GP Practice. Participants can update their current medication via the app.

### Participant timeline

**Fig. 1:**
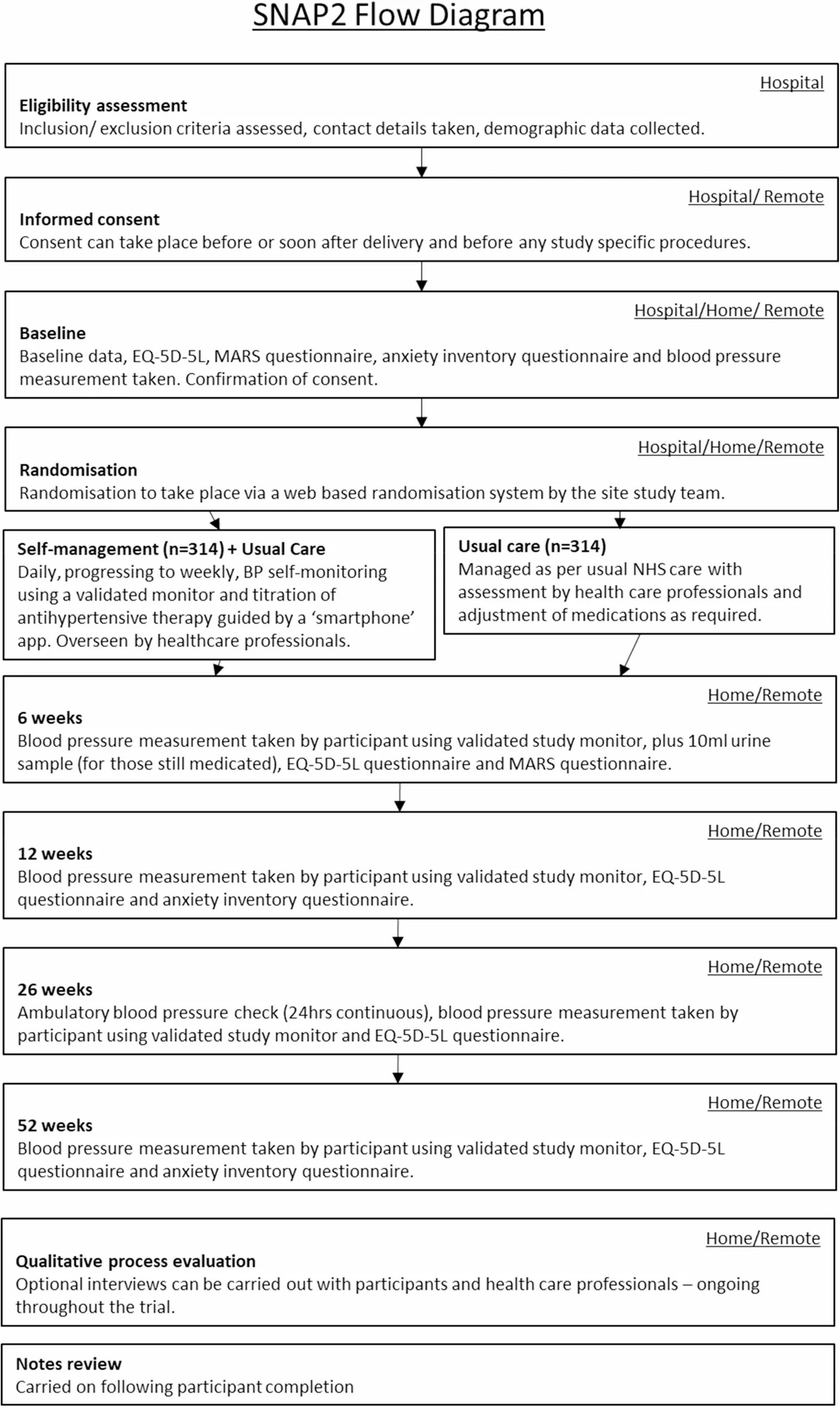
Participant flow

A flow diagram of the data collection tools and schedule (Fig. 1) represents the participant timeline for both study groups.

Contact details (to arrange follow-ups) and baseline demographics, medical history, obstetric history and social history will be collected at baseline. Participants will be asked to complete the EQ-5D-5 L health-related quality of life questionnaire, the Short Form Anxiety Inventory Questionnaire and Medication Adherence Report questionnaire.

Follow-up contacts (remote or in person) will occur at approximately 6 weeks, 12 weeks, 22–39 weeks and 48–60 weeks postpartum and involve repeated questionnaires (Fig. 1). The participant will be asked to take up to 6 (with a minimum of 3) blood pressure readings, 1 min apart with the provided, validated BP monitor. Participants who remain on antihypertensive medication at 6-weeks post-partum will be asked to obtain and post a 10 ml urine sample to test for adherence by measurement of urinary antihypertensive metabolites using LC- MS/MS at centralised core laboratory [26] (Supplementary file – SNAP2_Urine specimen collection and storage).

Participants will be asked to wear an ambulatory BP monitor for 24 h to collect the primary outcome measure at the 22–39 week time point.

A participant may choose to withdraw from the study at any time or investigators may withdraw a participant for reasons such as ineligibility overlooked at screening, or loss of capacity. For participants who withdraw from the study completely, their data and samples obtained up until the point of withdrawal will be retained for use in the analysis. No further data or samples will be collected after withdrawal. Participants may discontinue from the intervention but remain in the study for follow-ups. Reason for any withdrawal or discontinuation will be recorded in the eCRF.

### Methods: assignment of interventions

Participants will be randomised by research midwives at participating sites using a secure online system and allocated to intervention or control group on a 1:1 basis stratified by recruitment site with minimisation for principal diagnosis (chronic hypertension or gestational hypertension/pre-eclampsia) and mean postnatal diastolic blood pressure (last 3 readings in clinical record, < 83 mmHg vs. ≥ 83 mmHg) and number of anti-hypertensive medications prescribed (< 2 vs. ≥ 2 medications). The recruiting staff member will then inform the participants of allocation group and provide the appropriate participant pack.

Participants, recruiting staff and data management staff within the clinical trials unit will not be blinded to intervention group, due to the nature of the intervention.

### Methods: data management, sample size and analysis

#### Data management

A study-specific, secure, password-protected, eCRF data management system will be used to collect participant data from baseline and follow-up appointments. Data entry will be completed by recruiting staff with the option to be completed as an online survey by the participant if in person or if a participant is non-contactable by the research team, thus promoting follow-up rates. Paper CRFs will be used as a backup. External data, including ambulatory blood pressure monitoring (ABPM) results and laboratory analyses of urine samples, will be imported into the database. These data will be linked to participant records and stored securely. The system incorporates data entry and validation rules to reduce errors, with the aim of facilitating auditing and data quality assurance.

#### Sample size determination

Using data from the SNAP-HT feasibility trial and its follow-up study, it is estimated that the standard deviation of mean daytime ambulatory diastolic BP at 6 months will be 10 mmHg (inflated from 8 mmHg to take into account greater heterogeneity between recruiting sites in this multicentre trial). This means that 266 women (133 per randomisation group) are needed to detect ≥ 4 mmHg difference (standardised difference 0.4) with alpha 0.05 (2-sided) and 90% power. Assuming a 15% drop out during follow-up then 314 (157 per group) women will be required [16].

The aim will be to recruit approximately similar numbers of women with gestational hypertension/pre-eclampsia and chronic hypertension meaning that at least 628 women will be recruited overall. Assuming that the heterogeneity between these groups is small enough, 80% power will be retained for at least 5 mmHg difference in diastolic BP for sub-groups of at least 40% of the total sample size, e.g. white vs. non-white (expected 60:40 proportions). Furthermore, if appropriate to combine, the full sample of 628 women would allow smaller effect sizes to be detected (effect size = 0.26–90% power).

To achieve the required sample size, the trial will be undertaken in approximately 25 maternity hospitals in England. This will mean each site will be required to randomise 2 participants per month to meet the recruitment total. Previous work has shown that women are willing to participate in a self-management trial, suggesting the target will be achievable within the planned recruitment period.

Regular monitoring of demographic information of the trial sample will aim to highlight equity issues in recruitment that will be addressed promptly; aiming for a final sample that is representative of the participating sites and those affected most by hypertensive pregnancy.

#### Outcomes

The primary outcome of the trial will be the difference in mean daytime ambulatory diastolic blood pressure, measured at 22–39 weeks post randomisation, between groups, collected via an objective automated method (ambulatory BP monitoring). Secondary outcomes are listed in Table 2. Adverse events deemed to be related to the trial intervention or stroke, intracranial haemorrhage and seizure, will be recorded.

**Table 2:** SNAP2 Secondary outcomes and timepoint of evaluation

Blood pressure outcomes	
- Mean daytime ambulatory systolic BP	22–39 weeks
- Mean 24-hour and nocturnal ambulatory blood pressure (both systolic and diastolic)	
- Systolic and diastolic BP at follow-up (mean of 2nd /3rd readings and mean of 2nd -6th reading)	Baseline, 6, 12, 22–39 and 48–60 weeks
Quality of life outcomes	
- EQ-5D-5 L	Baseline, 6, 12, 22–39 and 48–60 weeks
- Short form anxiety inventory questionnaire	Baseline, 12 weeks, 48–60 weeks
Medication adherence outcomes	
- Prescence of urinary antihypertensive metabolites	6 weeks
- MARS questionnaire	Baseline, 6 weeks
Adverse event outcomes	
- All cause admissions	End of trial period
- Pregnancy hypertension admissions	
- Cardiovascular events	
Economic outcomes	
- Healthcare resource use and cost analysis of key cost drivers	Baseline, 6, 12, 22–39, 48–60
- Cost-consequence analysis presenting costs and key outcomes in a disaggregated manner	Over trial period
- Long term modelled cost-utility analysis using quality-adjusted life years (QALYs)	Projections modelled over woman’s lifetime
Qualitative process evaluation outcomes	
- The experiences and views of women and healthcare professionals on self-management and the interplay of an intervention within women’s lives and care pathways	Ongoing throughout trial period

#### Statistical methods

Participants will be analysed according to their randomised group assignment irrespective of the intervention they actually received or adherence of intervention. The primary analysis population will include all eligible participants for whom data is available. Participants who withdraw from the trial will be included in the analysis until the point at which they withdrew.

Study results will be reported in accordance with the CONSORT 2025 guidelines and statistical analysis plan prepared prior to recruitment start [27]. Descriptive statistics will be used to describe recruitment of the first 100 participants as part of the internal pilot [27].

Baseline variables will be presented by randomised group using descriptive summaries. There will be no tests of statistical significance nor confidence intervals for differences between groups on any baseline variables.

The primary estimand is the mean difference in daytime ambulatory diastolic BP at 22–39 weeks for all randomised participants, as defined by protocol eligibility criteria, regardless of what intervention they actually received or adherence of intervention. A generalised linear mixed-effects model utilising data collected at all time-points from randomisation will be fitted to the data with mean daytime diastolic BP measured at 22–39 weeks as the response variable, adjusting for minimisation factors and randomised group fitted as fixed effects and the model will include site as a random effect.

Missing data for the primary outcome will be handled using multiple imputation before fitting the mixed effect model.

A similar approach will be used for continuous secondary outcomes, and a generalised linear model for binary outcomes. Time effects will be included in the model for repeated measures outcomes where appropriate.

Safety outcomes will be compared using Fisher’s exact or Chi-square test. Pre-specified subgroup analyses of the primary outcome will be explored by baseline diastolic BP, principal diagnosis, ethnicity, indices of multiple deprivation quintiles, social status, and parity.

Missing data in all outcomes will be inspected and reported, with reasons given where available and missing data mechanism explored across randomised groups. We will explore the mechanism of missing data, though the mixed effects model implicitly accounts for data missing at random. Factors found to be predictive of missingness will be included as fixed effects in the analysis models.

Sensitivity analyses of the primary outcome will assess the robustness of the missing at random assumption required for mixed effect models. Additional sensitivity will assess mixed effects model including all available data of the primary outcome.

The level of significance will be set at 5% two-sided significance level. Where applicable, p-values will be adjusted for any multiple comparisons in order to maintain an overall type I error rate of 5%.

The final statistical analysis plan will be agreed prior to final data lock and prior to any analyses taking place. The analysis will be performed blind to the allocation group.

### Patient and Public Involvement (PPI)

Patient and public representatives have been involved throughout the SNAP2 programme to ensure the research reflects lived experience. Intervention and trial materials were developed with input from individuals with diverse backgrounds, including those disproportionately affected by hypertensive disorders. Two co-investigators are patient representatives. PPI members sit on the Trial Steering Committee (TSC) and data monitoring and ethics committee (DMEC), and maintain links with community and patient groups to support inclusive and appropriate trial procedures. They will contribute to interpretation of both qualitative and quantitative data, with attention to variation by ethnicity and deprivation, and help shape dissemination to reach underserved communities.

### Health equity

There remain stark gaps in maternity outcomes between women from Black, Asian, mixed and white ethnic groups and the SNAP2 trial seeks to understand and address these disparities with respect to blood pressure monitoring and care postnatally [7]. Working with co-applicants and PPI representatives, particularly from under-represented communities, those socially disadvantaged and minority ethnic groups, inclusive recruitment strategies have been devised aiming to ensure a diverse sample contributed to the intervention development, and are included in the trial and its evaluation. This includes targeted selection of NHS sites and engagement with community organisations. Ongoing assessment of recruitment approaches and regular monitoring of the trial sample will allow iterative changes in processes when needed. A study mobile phone will be available to ensure inclusion of those participants who may not own their own compatible device. The data collection for the qualitative process evaluation will be informed by purposive sampling to ensure the experiences and perspectives of women from socio-economically deprived and/or ethnically diverse groups are included.

### Qualitative process evaluation sub-study

An embedded qualitative process evaluation will aim to understand how postnatal self-management of blood pressure takes place and is integrated into women’s lives and clinical pathways. In-depth semi-structured interviews will assess both women’s and health professionals’ experiences of the trial.

A purposive sample of 30–40 participating women from either study arm, and up to eight women who decline participation, will be invited to participate in up to three longitudinal interviews. These will be offered as either in-person or remote interviews, scheduled with flexibility to suit the needs of the women and capture experiences throughout the trial period. Women will be recruited from across study sites, aiming for diversity in relation to location, age, socio-economic status, ethnic background, parity and medical history. 25–30 healthcare professionals with experience of the trial will be purposively sampled and offered either in-person or remote interviews. Interviews will last approximately 30–60 min.

Verbal or written consent will be obtained depending on the interview mode, and all participating women will be provided with a £20 voucher to thank them for their involvement. Additional support from translators, in-person or through telephone interpreting service and text relay will be offered to participants to support participation in the interviews.

Interviews will be recorded, transcribed verbatim and de-identified. Thematic analysis, conducted concurrently with data collection, will include open and more focused coding, constant comparison and exploration of deviant cases [28, 29]. Normalisation Process Theory and responsibilisation theories will be utilised to interrogate the data [30–32], providing a comprehensive understanding of how self-management becomes embedded (or not) in both women’s daily lives and healthcare professionals’ routine work.

### Health economics analysis

A within-trial economic evaluation in the form of a cost-consequences analysis will be conducted from an NHS perspective. At the individual participant level, data will be collected on antihypertensive medications prescribed and secondary sector healthcare contacts, using clinical notes review. Participant questionnaires at each follow-up point will be used to collect data on primary sector healthcare contacts. Additional work will estimate the resource use associated with the intervention. Unit costs from established national sources will be used to value healthcare resource use. An appropriate value set will be used to derive utility scores from the EQ-5D-5 L responses collected at each trial follow-up point [33].

Mean (standard deviation) resource use, costs, and quality of life will be reported for each arm of the trial. To compare arms, mean differences (95% confidence intervals) will be computed using regression methods adjusted for key baseline characteristics. Any missing data will be handled using multiple imputation techniques and results will be reported for the trial as a whole and for key sub-groups as specified by the trial’s clinical team. Given the evidence for future benefits from better blood pressure control post-pregnancy, health economic modelling will be utilised to extrapolate the longer-term costs and quality adjusted life years (QALYs) of any reduction in post-pregnancy blood pressure with the intervention during the trial. An existing Markov model which facilitates the modelling of longer-term cardiovascular events and their associated costs and consequences, will be adapted. Probabilistic sensitivity analysis will be used to handle uncertainty in the model’s input parameters and deterministic sensitivity analysis will explore the impact of varying the model’s time horizon. The model will be constructed to be able to simulate results for different sub-groups of women and will be developed and reported in accordance with good practice guidelines in the field [34].

### Data monitoring and audit

An independent DMEC will review the efficacy and safety data during the trial, an independent TSC will provide oversight. Composition of committees are detailed in the charter and available to share on request.

An internal pilot phase will be incorporated following recruitment of the first 100 participants, planned within the first 6 months to confirm trial feasibility. The decision to continue will be based upon a pre-specified criterion and decisions overseen by TSC and DMEC. The proportion of participants recruited from ethnically minoritised groups and areas of higher deprivation will also be reviewed regularly to ensure equitable representation, allowing for timely adjustments to recruitment strategies if needed.

A risk assessment and a Monitoring Plan will be in place and reviewed regularly and informed by the risk assessment, outcomes of any monitoring or changes to the protocol. Monitoring will be performed by the Primary Care Clinical Trials Unit Quality Assurance Manager or delegate.

Data will be evaluated for adherence with the protocol and accuracy in relation to source documents. Following written standard operating procedures, study staff with delegated monitoring responsibilities will verify that the clinical study is conducted and data are generated, documented and reported in compliance with the protocol, GCP and the applicable regulatory requirements.

### Dissemination

The results of this randomised controlled trial will be reported in peer-reviewed journals and presented at national and international conferences. Lay summaries and infographics will be available on the study website and social media platforms. Dissemination efforts will prioritise engagement with diverse audiences, leveraging existing collaborations with patient groups, charities, hospital sites, and community organisations, especially those serving ethnically minoritised and socioeconomically disadvantaged populations to ensure findings reach and benefit all relevant communities.

### Data management

A study-specific, secure, password-protected, eCRF data management system will be used to collect participant data from baseline and follow-up appointments. Data entry will be completed by recruiting staff with the option to be completed as an online survey by the participant if in person or if a participant is non-contactable by the research team, thus promoting follow-up rates. Paper CRFs will be used as a backup. External data, including ambulatory blood pressure monitoring (ABPM) results and laboratory analyses of urine samples, will be imported into the database. These data will be linked to participant records and stored securely. The system incorporates data entry and validation rules to reduce errors, with the aim of facilitating auditing and data quality assurance.

## Discussion

This manuscript describes the protocol of the SNAP2 study, a randomised controlled trial assessing whether postnatal self-management following hypertensive pregnancy leads to lower diastolic BP at 6 months postpartum compared to usual care. A qualitative process evaluation and economic analysis will inform acceptability of the intervention, our understanding of how self-management is implemented in women’s lives, clinical pathways and routine practices and the cost effectiveness and cost implications.

Results of the trial, process evaluation and economic analysis will be the culmination of the SNAP2 programme, providing vital data to inform care pathways for the management of postnatal hypertension, which is currently haphazard and sub-optimal. Importantly, the study emphasises equitable recruitment and analysis across diverse ethnic and socioeconomic groups to ensure the intervention’s relevance and accessibility for populations disproportionately affected by hypertensive disorders. Should self-management prove effective, incorporating this approach into clinical guidelines has the potential to improve both short- and long-term blood pressure control, thereby reducing lifelong cardiovascular risk and addressing persistent health inequalities.

## Supplementary Information

Supplementary Material 1.

## Data Availability

Following publication, trial data will be available from the authors upon reasonable request and with permission of the University of Oxford. Use of data will require approval by an independent review committee identified for this purpose, and investigators requesting data will need to provide a written protocol, including an analysis plan, and sign a data sharing/access agreement. Requests for Data Sharing should be directed to primdisc@phc.ox.ac.uk.
