# Supplementary material for: Self-management of blood pressure following hypertensive pregnancy (the SNAP2 trial): a prospective, multi-centre, unblinded, randomised controlled trial protocol
Source: BMC Cardiovasc Disord. 2026 Aug 4;26:1004. doi: 10.1186/s12872-026-06359-7 (PMC13721431; doi:10.1186/s12872-026-06359-7)
Supplement: Supplementary file 1 [file 12872_2026_6359_MOESM1_ESM.zip › SNAP2 Protocol Appendices/SNAP2_ICF_V1.0_08Feb2024.pdf]

# SNAP2

## Randomised Controlled Trial of Self-management of Postnatal Antihypertensive Treatment

**<Contact Details of Local Study team to be entered here:>**

**Participant ID: ________**

**INFORMED CONSENT FORM**

If you agree, please initial

| | | | |
|---|---|---|---|
| 1 | I confirm I have read and understood the participant information sheet version number ____________dated __ __ / __ __ __ / __ __ __ __ for the above study. I have had the opportunity to ask questions and had these answered satisfactorily. | | |
| 2 | I understand my participation is voluntary and that I am free to withdraw at any time, without giving any reason, and without my medical care or legal rights being affected. | | |
| 3 | I understand that even if I withdraw from the above study, data already collected about/from me will be used in analysing the results of the study. | | |
| 4 | I understand that relevant sections of my medical notes and data collected during the study and as part of study follow up may be looked at by authorised representatives from the University of Oxford, from regulatory authorities or from the NHS Trust, where it is relevant to my taking part in this research. I give permission for these individuals to have access to my records including those that identify me by name. | | |
| 5 | I agree to my General Practitioner or any other health care professional involved in my postnatal care being informed of my participation in the study, and to any necessary exchange of information about me between the study team and my GP. | | |
| 6 | I agree to supply my urine sample, as explained in the Participant Information Sheet. I understand that even if I withdraw from the above study, the sample collected from me may still be used in the study analysis. | | |
| 7 | I consider my study sample a gift to the Sponsor, the University of Oxford, and I understand I will not gain any direct personal or financial benefit from it. | | |
| 8 | I agree to take part in this study and undergo the study procedures as explained in the Participant Information Sheet. | | |
| | **OPTIONAL POINTS (not required for study participation)** | **YES** | **NO** |
| 9 | I wish to be provided with further information about taking part in the qualitative patient experience sub-study. I will then be able to make a decision as to whether or not I wish to take part in the sub study. (*optional*) | | |
| 10 | I consent that some of my identifiable data (such as NHS Number) will be used by the study team at the University of Oxford and NHS England for up to 10 years after the end of the study, so that I may be contacted for long term follow up study (*optional*) | | |

| | | | |
|---|---|---|---|
| 11 | I agree to be contacted about ethically approved future research studies for which I may be suitable. I understand that agreeing to be contacted does not oblige me to participate in any further studies (*optional*) | | |
| 12. | I agree for my urine sample to be used, in a form that does not identify me, in future research which has ethics approval. (*optional*) | | |

| | | |
|---|---|---|
| *Name of Participant* | *Date* | *Signature* |
| *Name of Person taking Consent* | *Date* | *Signature* |

When completed: 1 copy for participant; 1 for researcher site file (original); 1 copy to be kept in medical notes

**If you have any questions about this or any other aspect of the study please contact:**

SNAP2 Trial Team: snap2trial@phc.ox.ac.uk
