# Supplementary material for: Self-management of blood pressure following hypertensive pregnancy (the SNAP2 trial): a prospective, multi-centre, unblinded, randomised controlled trial protocol
Source: BMC Cardiovasc Disord. 2026 Aug 4;26:1004. doi: 10.1186/s12872-026-06359-7 (PMC13721431; doi:10.1186/s12872-026-06359-7)
Supplement: Supplementary file 1 [file 12872_2026_6359_MOESM1_ESM.zip › SNAP2 Protocol Appendices/SNAP2_RemoteConsentForm_V2.0_28Mar2024.pdf]

NUFFIELD DEPARTMENT OF PRIMARY CARE HEALTH SCIENCES — Primary Care Clinical Trials Unit

TRUST LOGO (if applicable), or a placeholder, 'local

Add contact details of the
local research team and
either the Chief or Local Investigator

Participant ID Number_________________________

CONSENT FORM

SNAP2

Randomised Controlled Trial of Self-management of Postnatal Antihypertensive Treatment

Researcher to seek and record informed oral consent, after participant has had sufficient time to think about whether they want to take part.

Please check the boxes to record that the question has been asked by the researcher and that the participant has responded in the affirmative:

1. Do you confirm that you have read the information sheet dated................... (version............) for this study? Have you had the opportunity to consider the information, ask questions and have these answered satisfactorily?	
2. Do you understand that your participation is voluntary and that you are free to withdraw at any time without giving any reason, without your medical care or legal rights being affected?	
3. Do you understand that even if you withdraw from the above study, data already collected about/from you will be used in analysing the results of the study?	
4. Do you understand that relevant sections of your medical notes and data collected during the study may be looked at by individuals from University of Oxford, from regulatory authorities and from the NHS Trust(s), where it is relevant to your taking part in this research? Do you give permission for these individuals to have access to your records?	
5. Do you agree to your General Practitioner or any other health care professional involved in your postnatal care being informed of your participation in the study, and to any necessary exchange of information about you between the study team and your GP?	
6. Do you agree to supply a urine sample, as explained in the Participant Information Sheet? Do you understand that even if you withdraw from the study above, the sample collected from you may still be used in the study analysis?	

7. Do you consider your study sample a gift to the Sponsor, the University of Oxford, and you understand you will not gain any direct personal or financial benefit from it?		
8. Do you agree to take part in this study and undergo the study procedures as explained in the Participant Information Sheet?		
**Optional:**		
9. Do you wish to be provided with further information about taking part in the qualitative patient experience sub-study? You will then be able to make a decision as to whether or not you wish to take part in the sub study. (*optional*)	Yes	No
10. Do you agree that some of your identifiable data (such as NHS Number) will be used by the study team at the University of Oxford and NHS England for up to 10 years after the end of the study, so that you may be contacted for long term follow up study (*optional*)	Yes	No
11. Do you agree to be contacted about ethically approved future research studies for which you may be suitable? Do you understand that agreeing to be contacted does not oblige you to participate in any further studies?	Yes	No
12. Do you agree for your samples to be used, in a form that does not identify you, in future research here or abroad, which has ethics approval? Do you understand this research may involve commercial organisations?	Yes	No

____

Name of Participant

___________________________ ________________ ________________________

Name of Researcher taking consent *Date* *Signature*

When completed: 1 for participant (e.g. emailed securely to participant); 1 for researcher site file (original); 1 to be kept in medical notes (if participant is a patient).
