# Supplementary material for: Self-management of blood pressure following hypertensive pregnancy (the SNAP2 trial): a prospective, multi-centre, unblinded, randomised controlled trial protocol
Source: BMC Cardiovasc Disord. 2026 Aug 4;26:1004. doi: 10.1186/s12872-026-06359-7 (PMC13721431; doi:10.1186/s12872-026-06359-7)
Supplement: Supplementary file 1 [file 12872_2026_6359_MOESM1_ESM.zip › SNAP2 Protocol Appendices/SNAP2_Urine specimen collection and storage.pdf]

Appendix – 6-week urine sample

Samples will be collected at home or in clinic and transferred to the laboratory at University Hospitals of Leicester via Royal Mail next day delivery. They will be stored up to 1 year after the end of the study for quality checking/verification, following which they will be stored for 5 years after the end of the study at a HTA licensed facility in case of future research, which participants will be asked to consent to.
