# Supplementary material for: Self-management of blood pressure following hypertensive pregnancy (the SNAP2 trial): a prospective, multi-centre, unblinded, randomised controlled trial protocol
Source: BMC Cardiovasc Disord. 2026 Aug 4;26:1004. doi: 10.1186/s12872-026-06359-7 (PMC13721431; doi:10.1186/s12872-026-06359-7)
Supplement: Supplementary file 1 [file 12872_2026_6359_MOESM1_ESM.zip › SNAP2 Protocol Appendices/SNAP2_PIS V4.0_19May2025_clean.pdf]

This study is funded by the NIHR

# The SNAP2 Study

*Blood pressure management following hypertensive pregnancy*

## A summary of the study

- This study is testing whether patients with high blood pressure can achieve better blood pressure control in the weeks and months following end of pregnancy, by using an app to enter their self-administered blood pressure readings (which are reviewed by a medical team) to adjust their medication accordingly.
- About half the people taking part in the study will be asked to monitor their blood pressure in an app daily until medication is withdrawn for up to 26 weeks, the other half will not self-monitor. All participants will continue to receive routine NHS care. **You will be randomly assigned to one of these two groups.**

## If you take part in this study:

**After Birth** → **6 Weeks** | **3 Months** | **6 Months** | **1 Year**

We will explain the study and ask for your consent following the end of your pregnancy. We will let you know if you're in the self-monitoring group or the usual care group

↓

We will call you at around 6 weeks, 3 months, 6 months and at 1 year to check everything is ok and to ask you to complete some questionnaires and take a blood pressure reading.

Please take your time to read this information and discuss it with others if you wish.

FUNDED BY NIHR | National Institute for Health and Care Research
UNIVERSITY OF OXFORD
NUFFIELD DEPARTMENT OF PRIMARY CARE HEALTH SCIENCES

# 1 Why are we doing this study?

Raised blood pressure is common in pregnancy (affecting approximately 1 in 10 women). Blood pressure remains elevated after birth, but usually returns to normal over 2-12 weeks.

**We would like to find out if home blood pressure management, using an app, might help to control blood pressure better than current procedures.**

# 2 Why am I being asked to take part?

You are invited to take part in the study as you are currently pregnant and have high blood pressure requiring treatment. We are looking to invite 628 participants to take part in the study.

# 3 Do I have to take part?

No. Taking part is entirely voluntary and will not affect your current or future NHS treatment. You can talk to your GP or midwife for independent advice about taking part.

# 4 What will happen if I take part?

You will be asked to sign a consent form to confirm that you agree to take part in the study. We will ask you some questions about your medical history, your pregnancy and day-to-day life. This will take around 30 minutes. You will then be randomly assigned by a computer to either Group 1 or Group 2. This is done randomly because this is the best way to do a fair comparison of the two groups.

Please see the next section for information specific to which group you are randomly allocated to and more details on follow up appointments.

Flexibility will be offered when booking, in terms of timings of follow-up appointments .

Additionally, you may be invited to take part in interviews. There is more information on this optional part of the study on a separate information sheet – please let us know if you may be interested in this.

## Usual care group

This means that you will carry on receiving your usual care as you did before the study from your midwife/consultant/healthcare professional. You will receive a blood pressure monitor and will have your blood pressure monitored by your community midwife and GP once you have been discharged from hospital .

Participation in the usual care group is very important to the research. We will follow you up at the end of the study in the same way as the people who are in the self-management group.

## Self-monitoring group

If you are in the self-management group, we will give you a blood pressure monitor to keep and show you how to use it. We will tell you how you can access the "My Blood Pressure Care" app and show you how to use it. You will receive an instruction leaflet with more details.

You will be asked to take your own blood pressure readings. This will involve taking 2 readings, 1 minute apart every day in the first few weeks. If the readings are raised, you may be asked for more readings.

An obstetrician or your GP will look over your blood pressure readings from the app and make any necessary changes to any blood pressure medication. You will receive a text message confirming any changes.

## What will happen if I have high or low blood pressure readings?

If you are in the self-monitoring group and the reading shows that your blood pressure is high or low, the app will send you clear instructions about what to do next, including who to contact and with what urgency.

Your readings will be reviewed by a healthcare professional, and if needed you may be asked to increase, decrease or stop the dose of your blood pressure medication. If this is the case, you will get a notification explaining the medication change.

If you are in the usual care group, please discuss this with your healthcare professional who will then advise on next steps.



This study is funded by the NIHR

# 7 Optional interviews

There is an optional linked patient experience study, where we will ask about your experience on the SNAP2 study. If you decline to take part in the SNAP2 study, you will be asked if you would like to take part in an optional interview to discuss why you declined and what you think about the self-management approach.

We will work with midwives to interview people from diverse groups. Please be aware that not everyone who expresses an interest to take part will be invited for an interview.

# 8 Who is organising and funding the research?

The research is being financed by the National Institute for Health Research. The sponsor is the University of Oxford, and the research is being managed by the Primary Care Clinical Trials Unit, at the University of Oxford's Department of Primary Care Health Sciences. The Chief Investigator for this study is Professor Richard McManus. Your doctor will not be paid for including you in this study.

# 9 How have patients and the public been involved?

Service users who have experienced high blood pressure during pregnancy have helped develop what research questions should be asked and the design of the patient-facing documents. They are involved in the trial management group and will continue to be involved in the study.

# 10 What if there are any problems?

If there is a problem or you have any concerns, you can contact the Trial manager (details at the end of this document).

The investigators recognise the important contribution that volunteers make to medical research, and will make every effort to ensure your safety and wellbeing.

The University of Oxford, as the research sponsor, has appropriate insurance in place in the unlikely event that you suffer any harm as a direct consequence of your taking part in this study.

In the event that something does go wrong and you are harmed during the research, and this is due to someone's negligence then you might have grounds for a legal action for compensation.

While the Sponsor will cooperate with any claim, you may wish to seek independent legal advice to ensure that you are properly represented in pursuing any complaint.

The study doctor can advise you of further action and refer you to a doctor within the NHS for treatment, if necessary. At any time during the study, you will be entirely free to change your mind about taking part, and to withdraw from the study. This will not affect your subsequent medical care in any way.

# 11 If you want to make a complaint

If you wish to complain about any aspect of the way in which you have been approached or treated in this study, you should contact the study team via the email address snap2trial@phc.ox.ac.uk or you may contact the University of Oxford's Research Governance, Ethics & Assurance Team (RGEA) on 01865 616480, or the director of RGEA at using the following email address: rgea.complaints@admin.ox.ac.uk

The Patient Advisory Liaison Service (PALS) is a confidential NHS service that can provide you with support for any complaints or queries you may have regarding the care you receive as an NHS patient. PALS is unable to provide information about this research study.

If you wish to contact the PALS team please contact

## Study Co-Ordinator contact details

For more information about the study please contact the trial manager, Ellie Newbury on 0808 2524539 or snap2trial@phc.ox.ac.uk

***Thank you for considering taking part!***

## More information (continued)

### What will happen to the samples I give ?

At 6 weeks after you start participating in the trial, we will ask you to take a urine sample at home and send it to a laboratory in the post. Urine samples will be analysed for medication adherence at the University of Leicester.

After the study analysis, any remaining material will be stored at a Human Tissue Act-licensed facility and with your consent, they may be used for future ethically-approved research. After this, they will be destroyed.

If you agree for your sample to be used in future research, they will be used in a form that does not identify you, but by ethically approved research projects may take place in hospitals, universities, non-profit institutions, or commercial laboratories worldwide. Because they will be shared in a form that does not link back to you, it will not be possible to withdraw them after they are shared.

### Will my taking part in this study be kept confidential?

Yes. All study records will be identified only by a code. We will only use personal identifiers such as names, date of birth and NHS numbers where this is necessary, e.g. to contact you to arrange follow up appointments.

The urine sample you provide will only be identified by your code and date of birth. Information that can identify you will only be held securely on a database. Only members of the study team or specific authorised individuals will have access to this database for the purposes of the study, or the purposes of monitoring and/or audit.

During the study, responsible members of the University of Oxford and the relevant NHS trusts may be given access to data for monitoring and/or audit of the study to ensure that the research is complying with applicable regulations.

If you agree to your data being kept to contact you regarding future follow up, this data will be kept for up to 10 years. If you don't agree to be contacted for future follow up, your contact details will be destroyed once your follow up appointments are complete.

You will be given a study number so that any information that you provide to us will not be directly identifiable .

## More information (continued)

### What will happen to my data?

Data protection regulation requires that we state the legal basis for processing information about you. Research is a task that we perform in the public interest. The University of Oxford, as sponsor, is the data controller. This means that we, as University of Oxford researchers, are responsible for looking after your information and using it properly. We will use the minimum personally-identifiable information possible.

The local research team will use your details such as name, NHS number, home address, email address and phone number to contact you about the research study follow-up appointments, and to contact you to advise if your medication needs changing.

Data from the app/text is stored securely behind NHS firewalls on NHS servers, which are owned by Oxford University Hospitals.

The local NHS Trust will use your name, NHS number, home address to contact you about the research study and to oversee the quality of the study. They will keep identifiable information about you from this study in accordance with their local policy. A copy of the consent form from this study will be kept in your medical records for as long as those records are retained.

If you agree to this, we will keep some identifiable information about you (such as your consent form, which identifies you by name, and NHS Number) for up to 10 years after the study has finished in order to contact you about further long term follow up. This will be dependent on funding .

Research data that is collected from the study will be kept for 20 years. Anonymised data may be shared with other researchers for research purposes. Data protection regulation provides you with control over your personal data and how it is used. When you agree to your information being used in research, however, some of those rights may be limited in order for the research to be reliable and accurate.

*Further information about your rights with respect to your personal data is available at https://compliance.web.ox.ac.uk/individual-rights.*

***You can find out more about how we use your information by contacting the trial manager.***





## Follow up visits

In addition to your usual care, we will arrange appointments to take place at 6 weeks, 3 months, 6 months and 1 year after the end of your pregnancy. These visits can either take place at the hospital or at home/remotely. If we can't get hold of you for your follow up visit, we will send you a reminder by post, text or email.

During these appointments, we will go through short questionnaires with you, and your blood pressure will be taken. These appointments will last between 30 minutes to an hour. We will ask you to provide a urine sample of 10ml (i.e. about a tablespoon) at 6 weeks and provide detailed instructions on how to do this. Your medical records may also be accessed, with your consent to collect information about you.

At the time of your 6 month appointment, we will ask you to wear a blood pressure machine that measures your blood pressure for 24 hours (an ambulatory blood pressure monitor). The study team will contact you by text, email, phone call and post to arrange delivery and return of the ambulatory blood pressure monitor. They will also explain what is involved in using the monitor. Once the monitoring is complete, we ask you to post the monitor back to us using a pre-paid envelope which we provide. If you are unable to post the monitor, we will arrange a courier to collect it. If you complete the 24 hour blood pressure monitoring, you will receive a £25 voucher.

## 5 Possible advantages and disadvantages of taking part

Taking part in the study may give you better information and understanding about your blood pressure. If possible, we hope that achieving better blood pressure control in the period after birth may contribute to better long-term health outcomes. However, the study is being conducted as it is not clear if medication adjustments (facilitated by the app and directed by clinicians' review) can achieve better blood pressure control than standard care.

We hope that information from this work will improve the care of women with raised blood pressure after birth in the future. We think there is very little risk of harm in taking part.

All women will still receive their usual care while in this study.

The only disadvantage is the extra time taken to measure blood pressure for women allocated to the self-management group and the additional time spent with the study team at the follow up visits. Other than the time taken to undertake the study, we do not anticipate any other disadvantage from taking part.

## 6 More information about taking part

### What happens if I change my mind?

If you do decide to take part but change your mind later, you are free to withdraw at any time. To do this, you can phone, write to or e-mail the Trial Manager on **0808 252 4539** or **SNAP2trial@phc.ox.ac.uk.** You do not need to give us a reason. This will not have any effect on your current or future NHS treatment. Any information you have given up to that point would still be used in the study results.

### Will my expenses be paid?

Most follow up appointments will be able to be completed over the phone. If you need to travel to any appointments just for the study then your travel expenses will be reimbursed.

### Will my GP be notified about my participation in this study?

Yes, we will send a letter to your GP to inform them that you are enrolled in the study and provide them with guidance for medication adjustments.

### Has anyone reviewed the study?

All research in the NHS is looked at by an independent group of people called a Research Ethics Committee, to protect your safety, rights, wellbeing and dignity. This study has been reviewed and was given a favourable opinion by REC.

### What will happen to the results of the study?

The intention is to publish the results of the study in a medical journal. The results may also be presented at medical conferences. You will not be identifiable in any reports or publications that arise from this research study. The results will also be published on our website at http://www.phctrials.ox.ac.uk and a study summary will be sent to you if you have agreed for your contact details to be retained by the study team.

*If you would like to refer to any of this information on our website, just scan the QR code and follow the link!*
